# AMS-YOLO: multi-scale feature integration for intelligent plant protection against maize pests

**DOI:** 10.3389/fpls.2025.1640405

**Published:** 2025-10-03

**Authors:** Leilei Deng, Di Fang, Aziz Ullah, Qi Hou, Helong Yu

**Affiliations:** ^1^ College of Information and Technology, Jilin Agricultural University, Changchun, China; ^2^ College of Computer Science and Technology, Changchun University of Science and Technology, Changchun, China

**Keywords:** maize pests, multi-scale feature fusion, attention mechanism, object detection, intelligent plant protection

## Abstract

**Introduction:**

As a major global food crop, maize faces serious threats from pests that significantly impact crop yield and quality. Accurate and efficient pest detection is crucial for effective agricultural management. However, existing detection methods demonstrate inadequate performance when addressing challenges including diverse pest morphologies, inter-species similarities, and complex field environments. This study introduces AMS-YOLO, an enhanced detection model based on YOLOv8n, to address these critical challenges in maize pest identification.

**Methods:**

To improve pest detection performance, we developed three synergistic modules specifically designed to address the identified challenges. First, the SMCA attention mechanism enhances target recognition within complex environmental settings. Second, an MSBlock multi-scale feature fusion module improves adaptability to pests across different growth stages. Third, an AMConv optimized downsampling strategy preserves subtle features necessary for distinguishing similar pest species. These architectural improvements were integrated into the YOLOv8n framework to create the AMS-YOLO model.

**Results:**

Experimental evaluation on a dataset comprising 13 common maize pests covering comprehensive developmental stages demonstrates the effectiveness of AMS-YOLO. The model achieved 90.0% precision, 89.8% recall, 94.2% mAP50, and 73.7% mAP50:95, surpassing the original YOLOv8n by 3.1%, 3.7%, 3.2%, and 4.0%, respectively. Comprehensive comparative experiments showed superior performance over existing detection methods including SSD, RT-DETR, and various YOLO variants. Deployment tests on Jetson Nano revealed that the model size is only 5.3MB, representing a 15.9% reduction compared to the original YOLOv8n, with 19.6% fewer parameters and 16% reduced computational requirements while maintaining low resource utilization.

**Discussion:**

The proposed AMS-YOLO model successfully addresses key challenges in maize pest detection through targeted architectural improvements. The lightweight design enables extended field deployment while maintaining high detection accuracy, making it highly suitable for resource-constrained agricultural environments. This advancement demonstrates significant potential for supporting more targeted pest management decisions, contributing to precision pesticide application and resource optimization in field conditions, thereby advancing intelligent and sustainable plant protection.

## 1 Introduction

Maize (*Zea mays* L.; corn) is a globally important food crop, animal feed, and industrial raw material (Nuss and Tanumihardjo, 2010; Erenstein et al., 2022), playing a critical role in ensuring food security and supporting industrial production. Maize suffers significant yield losses of up to 22.5% due to pests (Savary et al., 2019). These pests not only disrupt material transport during crop growth, but also act as vectors for viruses, ultimately leading to nutrient depletion, reduced quality, and lower yields (Ayres and Lombardero, 2018; Eigenbrode et al., 2018).

Traditional pest management relies primarily on chemical pesticides. The widespread application of chemical pesticides causes environmental pollution and reduced biodiversity (Sánchez-Bayo and Wyckhuys, 2019), and exacerbates pest resistance problems (Bass et al., 2015), with negative impacts on environmental and food safety becoming increasingly evident. Sustainable crop protection aims to effectively control pests while minimizing negative environmental impacts, protecting biodiversity, and supporting the long-term productivity and resilience of agricultural systems. This necessitates establishing enhanced, effective, and eco-friendly pest recognition systems to support decision-making for precision application and ecological pest management.

Pest identification technology has evolved from manual visual inspection or trap counting (Preti et al., 2021; Katranas et al., 2024), through traditional image processing techniques, to machine learning and deep learning approaches. Early studies mainly relied on manually extracted morphological features and shallow machine learning algorithms, including support vector machines (Suthaharan, 2016), adaptive boosting (Freund and Schapire, 1997), shallow artificial neural networks (Asefpour Vakilian and Massah, 2013), k-nearest neighbors (Wang et al., 2012), and ensemble methods (Larios et al., 2008). These algorithms rely heavily on manual feature engineering and often struggle to extract effective features in complex real-world environments. This leads to underfitting, low robustness, poor generalization ability, and high computational cost (Valan et al., 2019). Deep learning has fundamentally transformed pest identification. Object detection algorithms, particularly Faster R-CNN (Ren et al., 2015), SSD (Suthaharan, 2016), and the YOLO series (Redmon et al., 2016), have substantially enhanced the accuracy and robustness of pest detection. Two-stage detectors such as Faster R-CNN offer high precision but their computational complexity limits application in resource-constrained environments. Single-stage detectors including SSD and RetinaNet have improved speed by simplifying the detection process. The YOLO series, with its efficiency and real-time performance, has become the preferred solution for edge device deployment.

Nevertheless, existing models still face significant challenges when addressing the unique complexities of maize pest detection, failing to meet practical requirements of sustainable plant protection. These challenges primarily manifest in three critical areas. First, morphological diversity resulting from complete metamorphosis presents a major obstacle. Dramatic morphological variations across different developmental stages of the same species increase model generalization difficulty. Second, high similarity among pests of the same order or family substantially increases misidentification risk. Third, complex field backgrounds, including plant foliage, soil, and variable lighting and shadow conditions, further reduce detection precision. These three challenges severely limit the reliability and practicality of intelligent pest identification technology, constituting key technical barriers to achieving sustainable plant protection and precision agricultural management.

To address these challenges, this study proposes three key technical improvements based on the YOLOv8 framework:

1. enhancing the SMCA attention mechanism to strengthen the model’s capability for differentiating target regions within complicated backgrounds.
2. introducing the MSBlock attention mechanism to enhance morphological feature capture across different developmental stages.
3. designing AMConv to optimize the downsampling strategy, enhancing perception of subtle differences between similar pests.

**Figure 1:**
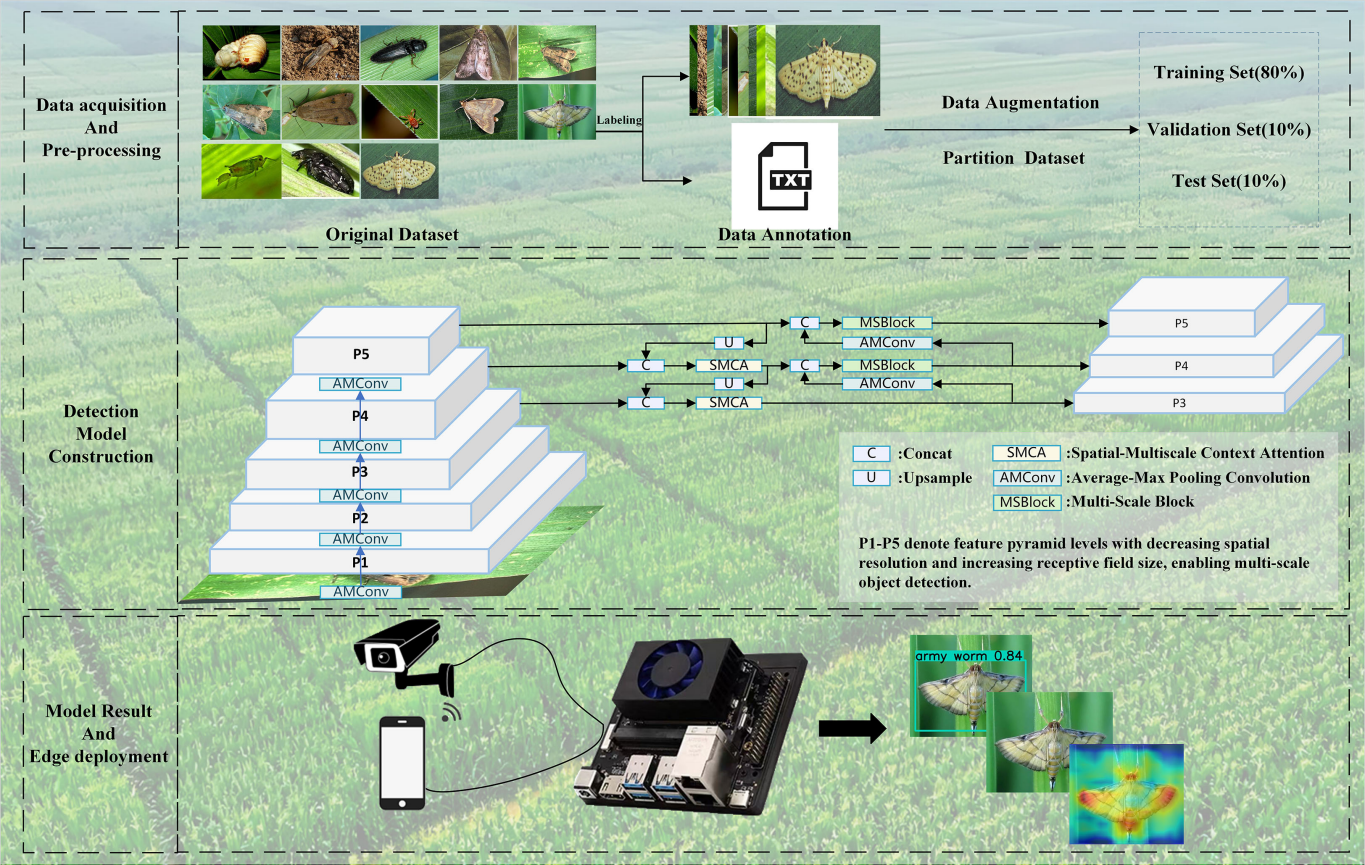
General framework of AMS-YOLO model training process, including data preprocessing, model architecture, and training pipeline.

**Figure 1** shows the overall framework of the AMS-YOLO model training process, including data preprocessing, model architecture, and the training pipeline. Experimental results demonstrate that the improved model substantially outperforms the original YOLOv8n across all performance metrics while maintaining real-time performance on edge devices, thereby offering effective technical support for sustainable plant protection.

## 2 Related work

Intelligent identification of agricultural pests is essential for precision plant protection. Previous studies have proposed various approaches to address the unique challenges in agricultural settings. (Jiao et al., 2022) proposed an adaptive feature fusion pyramid network for addressing insufficient multi-scale feature extraction in agricultural pest detection. This network introduces an adaptive enhancement module to minimize information loss in high-level feature maps. It obtained 77.0% accuracy with the AgriPest21 dataset, substantially outperforming methods such as SSD and RetinaNet. The model still demonstrates insufficient bounding box precision in complex scenarios. (Chen et al., 2023a) addressed misidentification among morphologically similar lepidopteran pest larvae through feature refinement methods, reaching 72.7% mAP on the SimilarPest5 dataset. The results demonstrate that model improvements for specific pest scenarios are more effective than general object detection methods.

Because of its excellent balance of speed and accuracy, the YOLO series has become the mainstream choice for agricultural pest detection. (Wang et al., 2025) introduced Insect-YOLO with Convolutional Block Attention Module (CBAM) for pest detection in low-resolution images, achieving 93.8% mAP50. (Zhang et al., 2022a) developed Coordination and Local Attention (CLA) mechanism and Grouped Spatial Pyramid Pooling Fast (GSPPF) module for pests with scale variations, reaching 71.3% mAP50 on a 24-class pest dataset. (Tian et al., 2023) proposed MD-YOLO with DenseNet blocks and Adaptive Attention Module (AAM) for small lepidopteran pests, achieving 86.2% mAP50. The pest species diversity remains limited. (Li et al., 2023) addressed the limited computational resources on edge devices. They proposed an improved point-line distance loss function and mixed online data augmentation algorithm. The method achieved 96.51% precision and 7.7ms detection time in passion fruit pest detection. This result demonstrates that single-stage detectors can be effectively deployed on edge computing devices. (Zhang et al., 2022b) integrated GhostNet with YOLOv5 to minimize redundant computation. Their approach improved mAP50 by 1.5% over original YOLOv5 in orchard pest detection while reducing parameters by 2–3 times. This provides a feasible solution for deployment on devices with limited resources.

Attention mechanisms, as a key technology for improving deep learning model performance, have demonstrated significant value in agricultural pest detection. (Tang et al., 2021) introduced squeeze-and-excitation attention for small and similar pests, achieving 71.6% mAP50 on a 24-class dataset. (Tang et al., 2023) improved Pest-YOLO by replacing the original SE mechanism with Efficient Channel Attention (ECA) mechanism. They combined it with transformer encoder to capture global features. Cross-Stage Feature Fusion (CSFF) was used to enhance small target representation. This improved detection capability for small pests, but the increased parameters limited inference speed (Liu et al., 2022). adopted triple attention mechanism (YOLOv4-TAM) and focal loss function to handle complex background and sample imbalance problems in tomato pest detection. The method achieved 95.2% average recognition accuracy. (Lv and Su, 2024) addressed the challenge of distinguishing similar diseases by combining Convolutional Block Attention Module with transformer encoder. The model achieved excellent performance in detecting visually similar apple leaf diseases. (Kang et al., 2023) addressed edge computing deployment challenges in rice pest detection. They proposed attention enhancement methods and knowledge distillation networks. (Cheng et al., 2017) used deep residual learning to solve pest identification problems in complex agricultural backgrounds. The method achieved 98.67% recognition accuracy, outperforming traditional methods.

Despite substantial advances in pest detection using deep learning, existing research still has four major limitations. First, dataset construction focuses on a single developmental stage or a specific environment. This makes it difficult for models to cope with morphological changes in different growth stages of the same pest. Second, detection algorithms insufficiently distinguish pests with high interclass similarity. Third, computational resources and detection accuracy remain poorly balanced. High-accuracy models are still difficult to deploy efficiently on agricultural edge devices. These limitations seriously constrain the practical value of intelligent pest identification technology in sustainable plant protection.

To address these limitations, this study implements targeted improvements to YOLOv8n through innovations in attention mechanisms, feature fusion, and multi-scale representation. Our goal is to build a high-performance pest detection system that can be implemented on resource-constrained edge devices while remaining robust in complex field environments, supporting sustainable agricultural goals of precision spraying and reduced pesticide use.

## 3 Materials and methods

### 3.1 Maize pest dataset

This study employed a maize pest identification dataset derived from the IP102 dataset (Wu et al., 2019), containing 13 maize pest species. After quality screening, we retained 4,293 valid images from the original dataset. We also captured 242 images using a Vivo S9 smartphone from a 2,500 m² experimental maize field at Jilin Agricultural University (43°79’N, 125°40’E) in July 2024. All images were annotated with rectangular bounding boxes under expert guidance. The dataset was randomly split into training, validation, and test sets in an 8:1:1 ratio.

**Figure 2:**
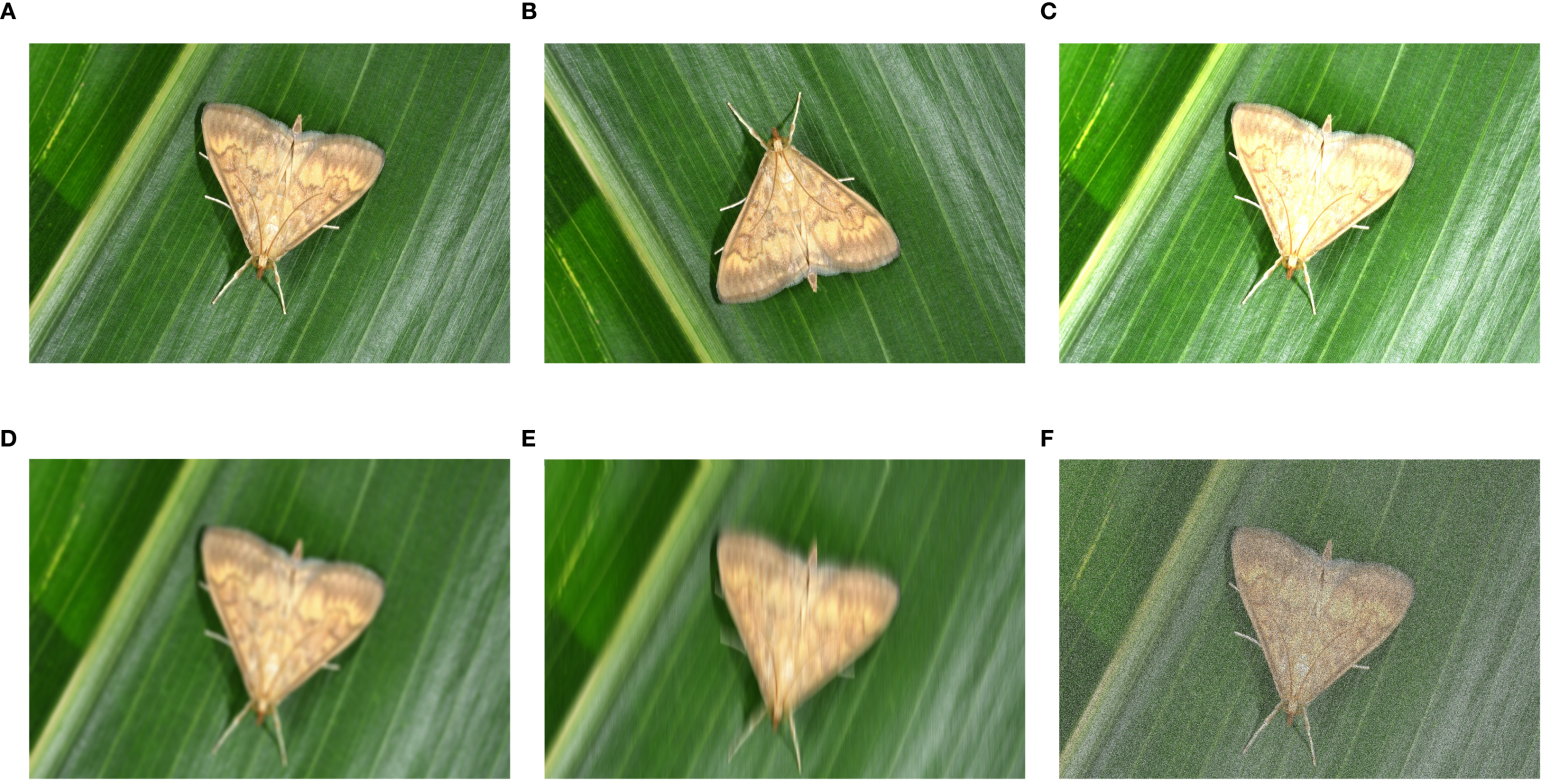
Comparison of different data augmentation techniques applied to maize pest images: **(A)** raw image; **(B)** vertical flip; **(C)** random brightness; **(D)** gaussian blur; **(E)** motion blur; **(F)** popcorn noise.

Sufficient and balanced training samples are critical for effective deep learning model training. The dataset covers 13 major maize pest species. These species exhibit notable morphological differences between developmental stages but high similarity among different species at similar stages. For example, *Ostrinia nubilalis* transforms from cream-colored larvae to winged moths. The three cutworm species *Agrotis ypsilon*, *Agrotis tokionis*, and *Agrotis segetum* exhibit nearly identical larval morphologies despite being different species. This ‘intra-class heterogeneity, inter-class similarity’ characteristic poses a major challenge to computer vision recognition systems. However, limited sample numbers and imbalanced class distributions may cause unstable model training and reduced generalization (Lu et al., 2025). Therefore, we implemented systematic data augmentation to optimize dataset quality (Tang et al., 2020). Five data augmentation methods are illustrated in **Figure 2**.

For the public dataset, we randomly applied one of five augmentation techniques: vertical flip, random brightness adjustment, Gaussian blur, motion blur, and Popcorn noise to increase data diversity. For self-collected field images, we applied three techniques: Gaussian blur, motion blur, and Popcorn noise. These methods simulate common imaging issues in agricultural environments, including camera shake, focus inaccuracy, and sensor noise. **Table 1** shows the dataset composition before and after augmentation by source.

**Table 1:** Datasets composition before and after augmentation by source.

Data source	Spilt	Original	Augmentation technique	Augmented
Public Datasets (IP102)	Training Set (80%)	3445	Choose one of the following methods at random: Vertical flip Random brightness Gaussian blur Motion blur Popcorn noise	6870
	Validation Set (10%)	429		858
	Test Set (10%)	429		858
Self-collected	Training Set (80%)	194	Using three data augmentation methods: Gaussian blur Motion blur Popcorn noise	776
	Validation Set (10%)	24		96
	Test Set (10%)	24		96

To ensure augmented image effectiveness, we inspected training set image quality, removing 183 images with minimal visual changes or poor quality. Considering validation and test sets are primarily used for model evaluation, we maintained the integrity of their augmented images to preserve evaluation consistency. The final dataset comprises 9,371 images, with detailed distribution shown in **Table 2**.

**Table 2:** Composition of training, validation, and testing datasets.

Name	Training set	Validation set	Test set
*Holotrichia diomphalia* (grub)	349	44	43
*Gryllotalpa unispina* (mole cricket)	695	87	87
*Pleonomus canaliculatus* (wireworm)	339	42	43
*Euxoa oberthuri* (white margined moth)	313	39	39
*Agrotis ypsilon* (black cutworm)	720	90	90
*Agrotis tokionis* (large cutworm)	489	61	61
*Agrotis segetum* (yellow cutworm)	621	78	77
*Tetranychus truncatus* (red spider)	512	64	64
*Ostrinia nubilalis* (corn borer)	678	85	85
*Mythimna separata* (army worm)	659	82	83
*Sitobion avenae* (aphids)	701	88	87
*Protaetia brevitarsis* (White-spotted flower chafer)	650	81	81
*Dichocrocis punctiferalis* (peach borer)	771	96	97

The “Name” column presents Latin scientific names of pest species with their corresponding common names in parentheses.

### 3.2 AMS-YOLO model structure

YOLOv8n builds upon its predecessor’s advantages and improves performance through architectural innovation and algorithm optimization. Its anchor-free detection framework abandons traditional preset anchor box limitations and directly predicts object location and size. The network backbone adopts an optimized CSPNet variant. It creates efficient feature extraction paths through the C2f module and enhances multi-scale target sensing capability with the SPPF module.

The feature fusion stage integrates FPN (Lin et al., 2017) and PAN (Li et al., 2018) structures, enabling bidirectional feature flow and complementary information interaction. The detection head utilizes a task decoupling strategy to separate classification and regression paths, optimizing their respective performances. For the loss function, Binary Cross-Entropy loss handles classification, while CIoU loss ensures accurate bounding box predictions for localization. The model incorporates a Task Aligned Assigner (Feng et al., 2021) to dynamically evaluate sample quality and balance classification accuracy with localization precision, achieving optimal speed-accuracy trade-offs.

**Figure 3:**
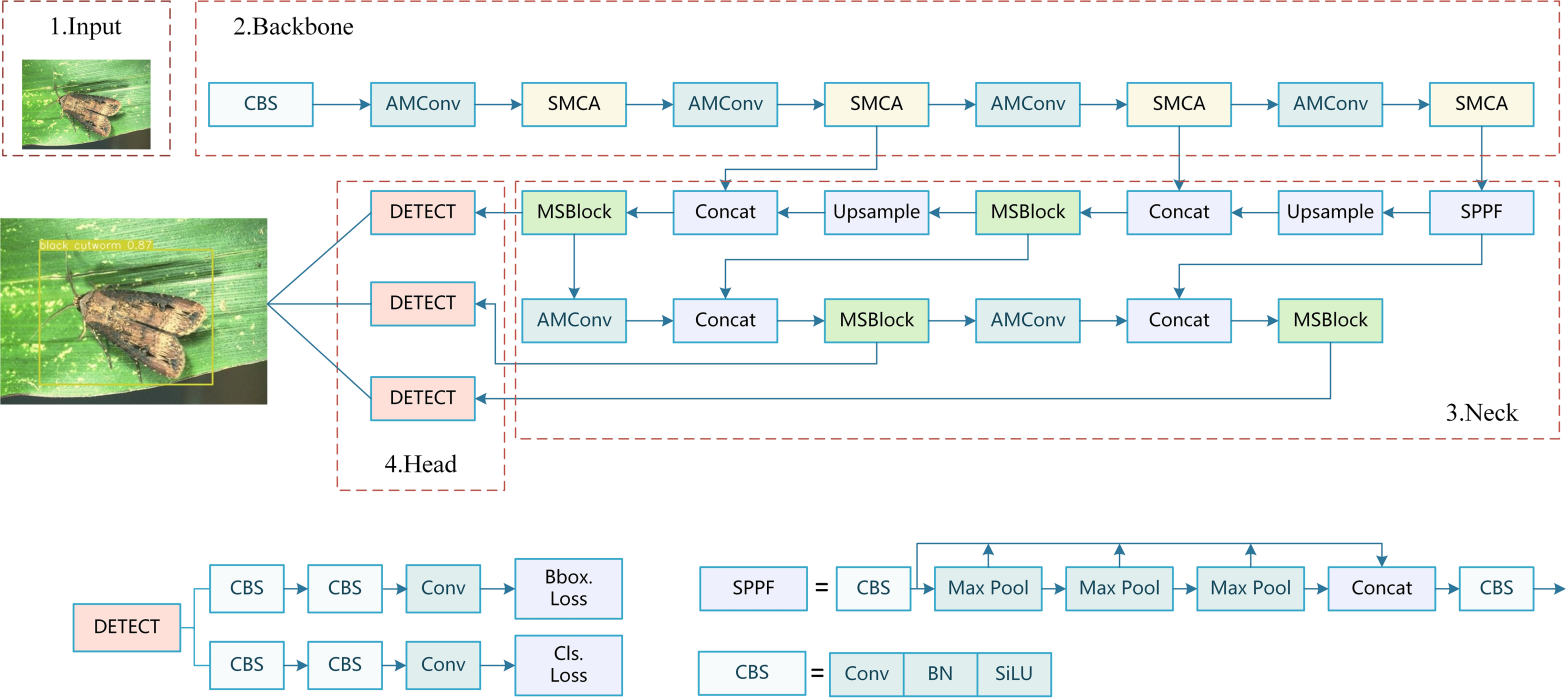
The complete framework of our AMS-YOLO model. AMConv is average max pooling convolution, SMCA is proposed spatial-multiscale context attention, MSBlock is multi-scale block, SPPF represents spatial pyramid pooling, Upsample refers to upsampling, Concat represents tensor concatenation, MaxPool2d represents maximum pooling, and DETECT serves as detection head.

However, despite YOLOv8’s robust capabilities for most detection applications, this model exhibits substantial constraints when applied to maize pest detection in real agricultural environments. These practical constraints substantially impact pest management decision reliability, ultimately affecting sustainable plant protection strategy effectiveness. To address these challenges, this study proposes AMS-YOLO, an improved YOLOv8n version specifically designed for agricultural pest detection applications. As illustrated in **Figure 3**, our framework processes maize pest image data through carefully designed architectural innovations.

In the AMS-YOLO model, the improved SMCA module enhances spatial-multiscale context awareness by introducing adaptive spatial weighting and multi-scale feature enhancement. The novel AMConv module preserves critical morphological details through adaptive kernel weighting and residual feature preservation. Additionally, the MSBlock embedded in the neck structure implements stage-aware attention and adaptive feature fusion. These targeted improvements work synergistically to enhance detection precision while maintaining computational efficiency suitable for edge deployment in agricultural settings.

#### 3.2.1 Spatial-multiscale context attention

Identifying pests in complex agricultural environments requires rich contextual information to avoid confusion between similar species and reduce background interference. However, inherent local convolution operations and excessive pooling challenge the extraction of sufficient global context and effective target detection. To address this, this study proposes the Spatial-Multiscale Context Attention (SMCA) module, which is incorporated within the backbone feature extractor of the YOLOv8n detection network.

**Figure 4:**
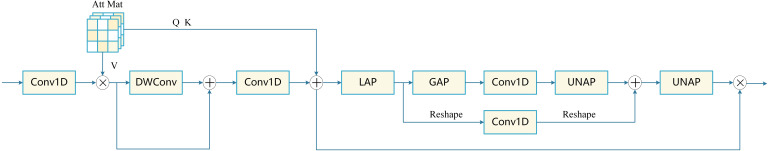
Structure of SMCA model.

The design of the SMCA module is based on two key observations. First, pest appearance in images is influenced by surrounding elements such as maize leaves and stalks, requiring full global context capture. Second, distinguishing morphological features of similar pests requires extracting local details. Unlike sequential attention mechanisms such as CBAM (Woo et al., 2018) or channel-focused approaches like ECA (Wang et al., 2020), SMCA employs a unified framework that simultaneously processes spatial self-attention and multi-level contextual information through joint local-global weighting, addressing the limitations of independent attention processing for morphologically similar pest identification. The SMCA structure is shown in **Figure 4**.

The core of the SMCA module consists of two key steps: spatial attention computation and multi-scale feature fusion. The spatial attention module first computes attention features using the multi-head self-attention mechanism, as shown in Equation 1:


(1) $$\begin{array}{c} X_{s}=Reshape(softmax(\frac{QK^{T}}{\sqrt{d}})V) \end{array}$$

Where Q , K , $V\in {\mathbb{R}}^{\left(h\times N\times d\right)}$ are the query, key, and value matrices, respectively. Here, *h* represents the number of attention heads, N=H×W indicates the total number of spatial positions, where *H* and *W* correspond to the height and width of the input feature map, respectively. The scaling factor $\sqrt{d}$ prevents extremely large values after dot-product, stabilizing the training process. The attention mechanism computes the similarity between *Q* and *K* to generate an attention map, which is then used to weight V , resulting in an enhanced spatial feature representation.

Following spatial attention, the SMCA module introduces a multi-level contextual attention mechanism (Wan et al., 2023), consisting of two branches, local and global, as defined in Equations 2 and 3:


(2) $$\begin{array}{c} F_{l}=Avgpooling(X_{s})\in R^{C\times k_{s}\times k_{s}} \end{array}$$

(3) $$\begin{array}{c} F_{g}=Avgpooling(F_{l})\in R^{C\times 1\times 1} \end{array}$$

Where $k_{s}$ determines the spatial range of local feature extraction, with a default value of 5. *C* denotes the channel count within the feature map. This $AvgPool_{local}(\cdot )$ applies adaptive average pooling to reduce spatial dimensions to $k_{s}\times k_{s}$ , while $AvgPool_{global}(\cdot )$ further compresses $F_{l}$ to a single value per channel. This two-branch strategy enables the module to concurrently process both local structural details and global contextual information.

To extract meaningful channel relationships from both $F_{l}$ and $F_{g}\;$ , a *1D* convolution operation with an adaptively determined kernel size k is applied to both local and global features. The feature maps are first reshaped to *1D* representations suitable for channel-wise convolution. $Conv1D_{k}(\cdot )$ applies one-dimensional convolution across channels to model inter-channel dependencies efficiently. The kernel size k is computed based on the number of channels C in the feature map using Equation 4:


(4) $$\begin{array}{c} k=[\frac{\log_{2}(C)+b}{\gamma }] \end{array}$$

Where b and γ are hyperparameters that control the receptive field size, with default values b=1 and γ=2 . The convolution kernel size k is maintained as an odd number, thereby preserving spatial symmetry in feature mapping. The kernel size increases logarithmically with the number of channels, enabling the attention mechanism to adaptively adjust its receptive field according to different feature dimensions. After applying $Conv1D_{k}$ to both $F_{l}$ and $F_{g}$ , the results are transformed by sigmoid function σ and combined to compute the final attention weight:


(5) $$\begin{array}{c} A_{final}=\omega \sigma (Conv1D_{k}(F_{l}))+(1-\omega )\sigma (Conv1D_{k}(F_{g})) \end{array}$$

Where ω is a learnable parameter with default 0.5 that balances the contributions of local and global attention, and σ is the Sigmoid activation function that transforms attention values to range [0,1], making them suitable for feature modulation. This balanced approach allows the model to adaptively focus on different levels of contextual information.

Finally, the computed attention weights $A_{final}$ from Equation 5 are applied to the input feature map $X_{s}$ from Equation 1 through element-wise multiplication, as shown in Equation 6:


(6) $$\begin{array}{c} Y=X_{s}⊙A_{final} \end{array}$$

Where ⊙ denotes element-wise multiplication. This operation performs channel-wise feature recalibration, emphasizing important features while suppressing less informative ones, resulting in a final enhanced feature map Y that effectively combines spatial attention with multi-scale contextual information.

**Figure 5:**
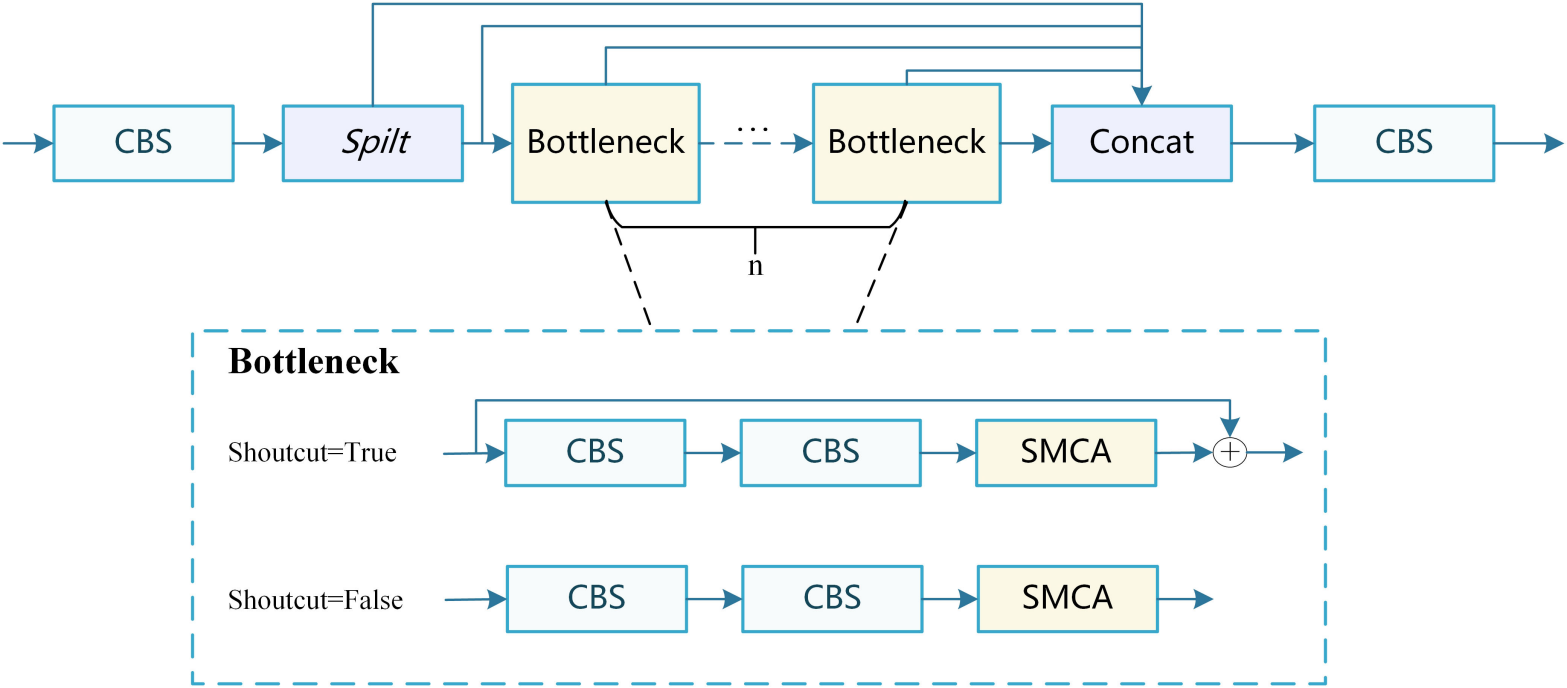
Architectural diagram of the C2f_SMCA module.

In AMS-YOLO, this study combines the SMCA module with the C2f structure to create the C2f_SMCA module, as shown in **Figure 5**. The C2f structure uses a multi-branch design, where one branch retains the original information, and the other is processed through a Bottleneck layer before merging.

Embedding SMCA into C2f fully leverages the complementary strengths of both components. The multi-branch structure of C2f provides a fundamental channel for feature extraction and propagation, while SMCA enhances feature representation through the attention mechanism. Additionally, the skip-connection structure of C2f facilitates gradient backpropagation, ensuring effective training of the SMCA module in deeper layers. This integration prevents feature information loss, offers an improved feature fusion mechanism, and maintains high computational efficiency. The multi-branch design also reduces the model’s reliance on individual features, thereby enhancing its robustness.

#### 3.2.2 Average-max pooling convolution

In target recognition tasks, downsampling operations are commonly used to reduce feature map size. However, the original YOLOv8n model’s downsampling operations cause detailed information loss when reducing feature map dimensions. This particularly impacts detection performance in complex backgrounds and for objects with varied scales.

Conventional downsampling approaches in CNNs typically use single-strategy processing. They employ either stride convolutions directly reducing spatial dimensions or pooling operations summarizing local regions. Stride convolutions allow learnable downsampling but may cause information loss. Pooling operations provide fixed downsampling with limited adaptability. Recent efficient convolution variants reduce computational constraints differently. GhostConv (Han et al., 2020) generates redundant feature maps through lightweight linear operations, reducing computational cost. Depthwise separable convolutions (Chollet, 2017) process each channel independently before combining them via pointwise convolution. These approaches, however, still operate through single-path processing strategies. Our proposed AMConv module uses a dual-path architecture combining average and maximum pooling strategies to preserve both global context and local detail during downsampling. To address this, we propose the Average-Maximum Pooling Convolution (AMConv) module by optimizing the convolution operations in YOLOv8n.

**Figure 6:**
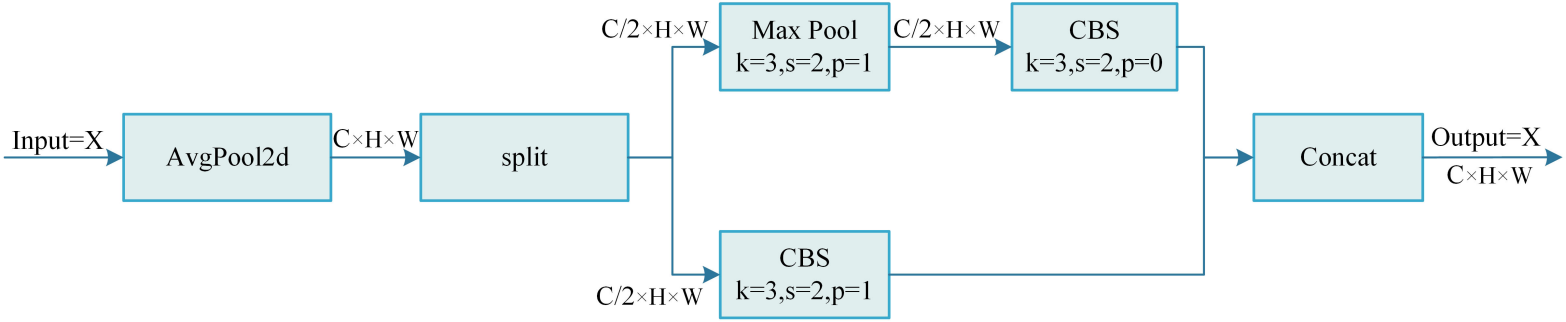
Structural diagram of the AMConv module.

The working principle of the AMConv module is illustrated in **Figure 6**. First, the input feature map undergoes average pooling with stride 1 and kernel size 2, preserving important global information while initiating downsampling. Next, the feature map is split along the channel dimension into two equal parts. The first part is processed using 3×3 convolution with stride 2 to extract features and reduce dimensionality. Meanwhile, the second part undergoes max pooling with 3×3 kernel and stride 2, followed by 1×1 pointwise convolution to enhance its nonlinear feature representation. Finally, the processed feature maps from both paths are concatenated along the channel dimension to form the AMConv module output.

#### 3.2.3 Multi-scale block

YOLOv8n introduces a large number of C2F modules throughout its neck structure for improving feature extraction. Nevertheless, such high complex computations and substantial parameter count associated with this design significantly increase inference time. Therefore, the Multi-scale Block with large kernel convolutions (Chen et al., 2023b) is introduced into the architecture. We adapt the original design by removing the Global Query Learning mechanism to reduce computational complexity while maintaining hierarchical multi-scale feature extraction capability. This makes the design more suitable for real-time pest detection applications. The streamlined MSBlock design enriches feature extraction by providing larger receptive fields, thereby enhancing the model’s contextual understanding capability for accurate pest detection.

**Figure 7:**
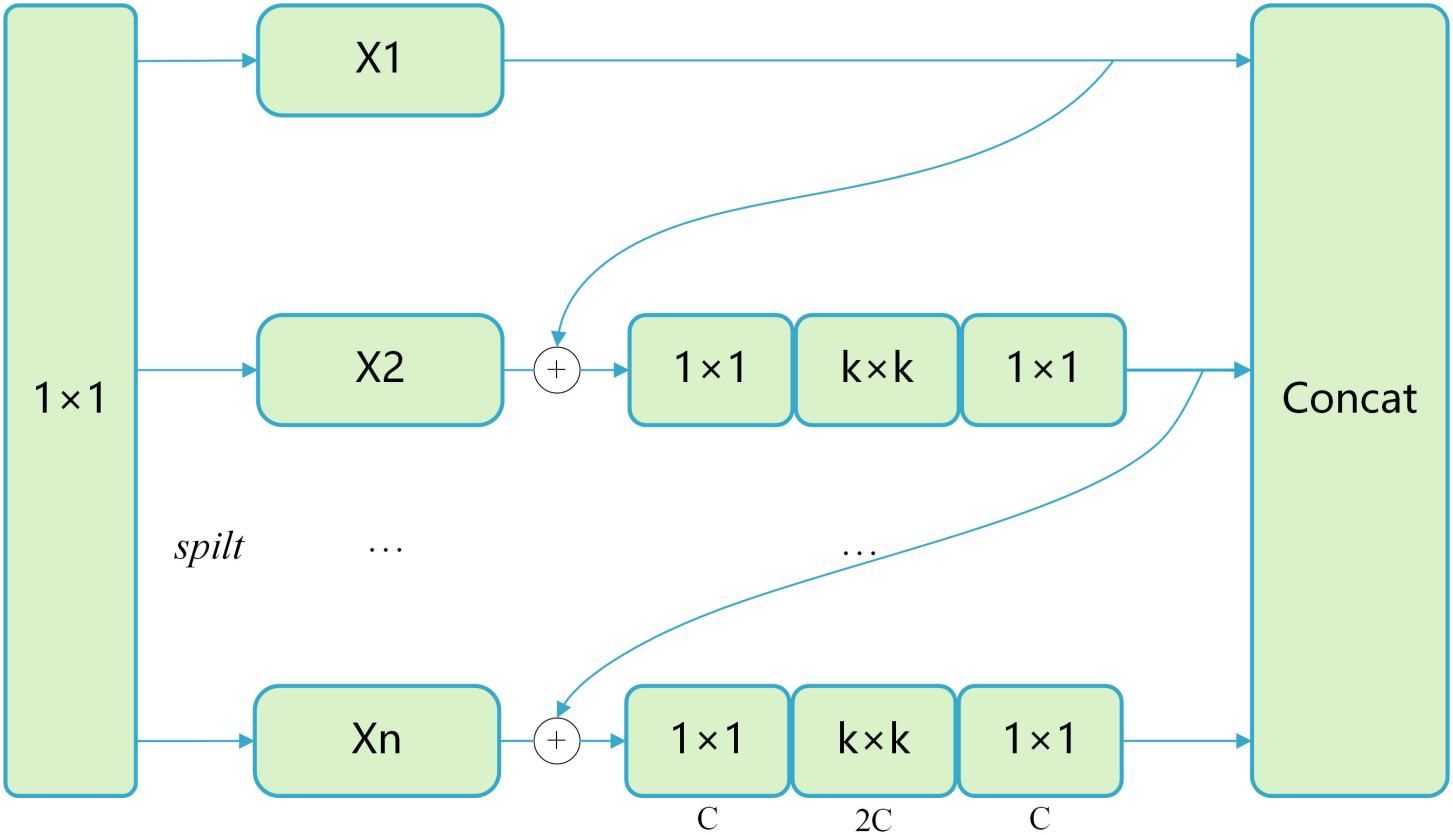
Multi-scale block structure diagram.

Specifically, the core idea is to split the input channels into several channel groups and then perform multi-scale convolutional operations, such as 1×1 , 3×3 , and 5×5 , on these sub-channels to improve the perception of targets at different scales. Efficient aggregation and feature enhancement of the channels are achieved through layer-by-layer convolution operations, while feature compression and fusion are accomplished using 1×1 convolutions to reduce computational overhead. The specific architecture and implementation of the MSBlock module are shown in **Figure 7**.

Suppose X represents the input feature map with C input channels. After a 1×1 convolution, the channel dimension of X is expanded according to the designed expansion mechanism. To balance computational efficiency and feature representation capability, we set the configurable hyperparameters expansion ratio $r_{expend}=3$ and down-sampling ratio $r_{down}=2$ . The expanded channel dimension is calculated as shown in Equation 7:


(7) $$\begin{array}{c} C_{expanded}=\frac{C\times r_{expend}}{r_{down}} \end{array}$$

The 1×1 convolution achieves dimensionality increase by setting the number of output channels to 1.5C , which is greater than the number of input channels C . In our implementation, the 1×1 convolution layer maps from C input channels to $C_{expanded}=1.5C$ output channels, effectively expanding the feature representation space before multi-scale processing.

The expanded feature map is then divided into *N* different groups, denoted as $\left\{X_{1},X_{2}\ldots ,\;X_{\text{n}}\right\}$ , where i ∈ [1,N] . The input is split into multiple branches, with each handling a different subset of channels. Each group processes features through inverted bottleneck layers with different kernel sizes. These kernel sizes include 1×1 , 3×3 , and 5×5 convolutions. Groups with kernel size k=1 use identity mapping for computational efficiency. Starting from the second group, each group’s input incorporates the output from the previous group. This establishes a cumulative feature propagation mechanism. This mathematical representation for the output $Y_{\text{i}}$ can be expressed as:


(8) $$\begin{array}{c} Y_{i}=\{\begin{array}{c} X_{i},\;\;i=1\\ IB_{k\times k}(Y_{i-1}+X_{i}),\;\;i>1 \end{array} \end{array}$$

According to Equation 8, the cumulative connection mechanism allows each branch to retain information from previous processing stages while incorporating new transformations. Within each branch, feature transformation is performed using a 1×1 convolution for channel expansion. This is followed by a k×k depthwise separable convolution and finally a 1×1 convolution for channel compression. All branches are then concatenated. A final 1×1 convolution is applied to facilitate interaction between the branches, with each branch encoding features at different scales.

By replacing the traditional C2F module with C2f_MSBlock on these P3, P4, and P5 feature levels within the neck architecture, this design enables such the model to focus on small-scale pest targets through the P3 layer features, enhancing the ability to capture fine details; medium-scale pest targets are captured by the P4 layer features, which focus on morphological characteristics; and large-scale pest targets are detected by the P5 layer features, utilizing large receptive field convolutions to ensure target integrity. This approach not only addresses the scale inconsistency problem in maize pest detection but also enriches the model with more detailed pest micro-features and improves localization accuracy for pest detection.

## 4 Experiments and analysis of results

### 4.1 Experimental setup and parameter configuration

The experiments used an NVIDIA RTX 4070 SUPER GPU and Intel Core i5 processor. The software environment used PyTorch deep learning framework with CUDA acceleration. **Table 3** provides detailed hardware and software configuration parameters.

**Table 3:** Experimental test platform configuration.

Hardware	Model number	Parameters
Operating System	Windows 11	RAM: 64 GB
CPU	Intel Core i5-13600KF	Frequency: 3.50 GHz
GPU	NVIDIA RTX 4070 SUPER	Video memory: 12 GB
Deep Learning Framework	PyTorch	Version: 1.12.1
Computational Platform	CUDA	Version: 11.3
Software environment	Python	3.9

For model training, we used the AdamW optimizer (Loshchilov, 2017) for stochastic gradient descent. **Table 4** shows other default hyperparameter settings.

**Table 4:** Training parameters.

Hyperparameters	Value
Image size	640 × 640
Epoch	200
Batch Size	16
Workers	4
Optimizer	AdamW
Learning Rate	0.002
Momentum	0.937
Weight Decay	0.0005

### 4.2 Dataset and evaluation metrics

This study selected the following evaluation metrics: Precision (P), Recall (R), mAP50, mAP50:95, Parameters, and Weights. These metrics assess the model’s performance in maize pest detection. mAP50 represents the mean average precision (mAP) at an IoU threshold of 0.5, while mAP50:95 denotes the average mAP across IoU thresholds from 0.5 to 0.95 (step size: 0.05). Equations 8–12 define these metrics.


(9) $$\begin{array}{c} Precision(P)=\frac{TP}{TP+FP} \end{array}$$

(10) $$\begin{array}{c} Recall(R)=\frac{TP}{TP+FN} \end{array}$$

(11) $$\begin{array}{c} AP_{i}=\int_{0}^{1}P_{i}(R_{i})dR_{i} \end{array}$$

(12) $$\begin{array}{c} mAP=\frac{1}{N_{c}}\sum_{i=1}^{N_{c}}AP_{i} \end{array}$$

(13) $$\begin{array}{c} Parameters=O(\sum_{i=1}^{n}(M_{i}^{2}\cdot K_{i}^{2}\cdot C_{i-1}\cdot C_{i})) \end{array}$$

In Equations 9 and 10, True Positive (TP) refers to positive samples correctly classified as positive. False Negative (FN) represents positive samples incorrectly classified as negative, and False Positive (FP) indicates negative samples incorrectly classified as positive. In Equations 11 and 12, n is the number of pest species. $P_{i}$ represents the precision of the i-th pest category, and $R_{i}$ represents its recall. In Equation 13, O denotes the order of magnitude, K represents the kernel size, C is the number of channels, M is the input image size, and i is the number of iterations.

### 4.3 Comprehensive performance analysis of model improvement and attention mechanisms

#### 4.3.1 Comparative experiments between the original and improved model

**Figure 8:**
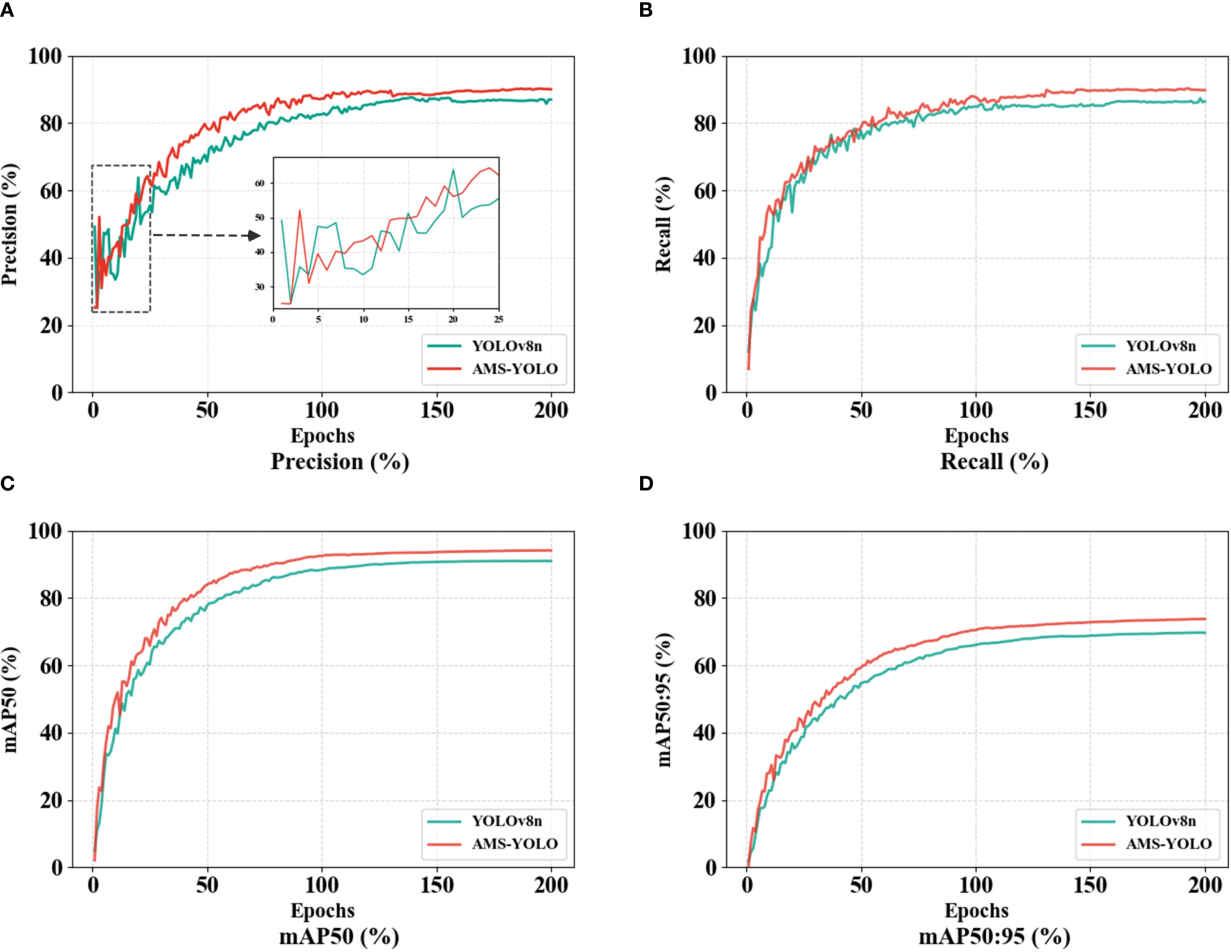
Training process curves of AMS-YOLO and YOLOv8n.

The Precision, Recall, mAP50, and mAP50:95 for the original YOLOv8n model and the AMS-YOLO model after 200 training iterations appear in **Figure 8**. In the early training stages, both models show relatively low performance metrics with significant fluctuations. This reflects gradual model learning of target features. As training progresses, the AMS-YOLO model demonstrates faster convergence and superior performance across all metrics. The AMS-YOLO advantage in mAP50:95, a comprehensive evaluation metric, is particularly pronounced, confirming its robustness across varying detection thresholds. By training completion, the AMS-YOLO model outperforms the original YOLOv8n model in all metrics, validating that the proposed improvement strategy successfully improves the detection capability of the model for maize pests.

**Figure 9:**
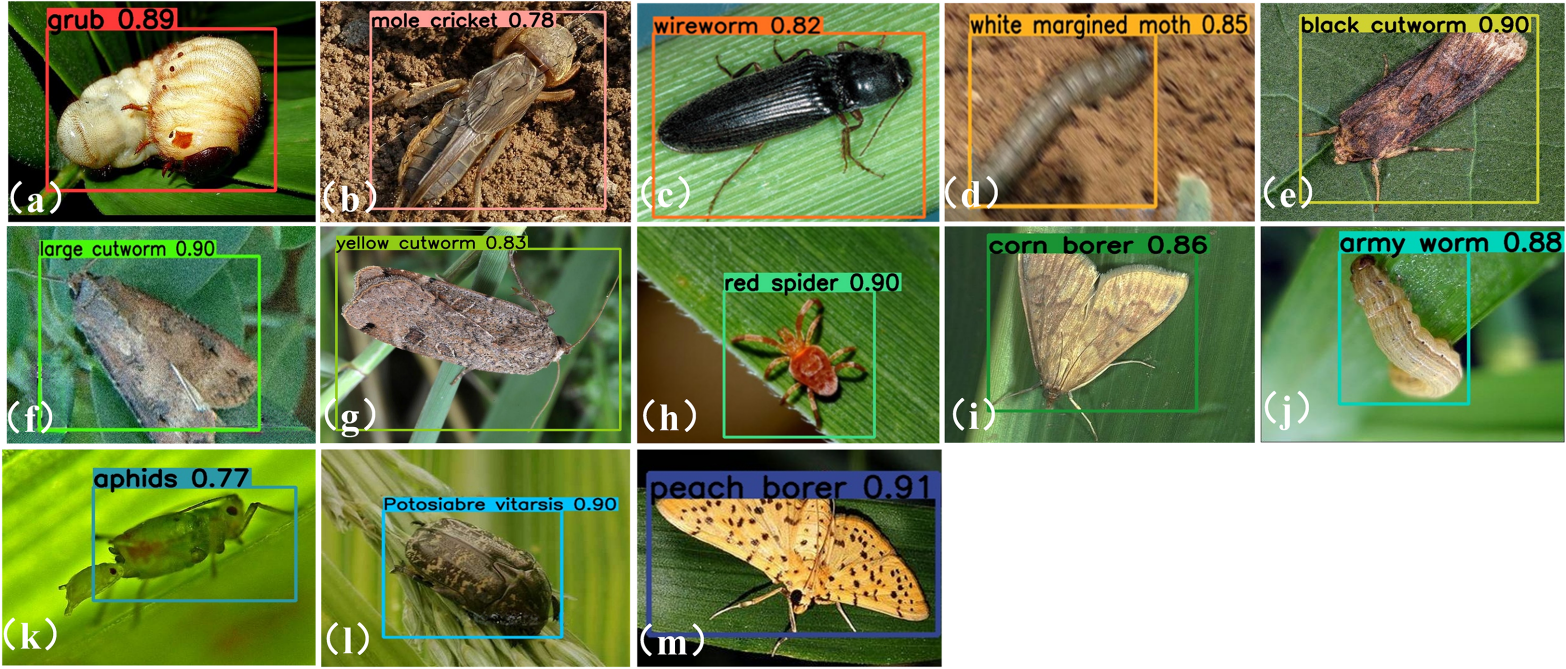
Visual result map. **(a)** grub **(b)** mole cricket; **(c)** wireworm; **(d)** white margined moth; **(e)** black cutworm; **(f)** large cutworm; **(g)** yellow cutworm; **(h)** red spider; **(i)** corn borer; **(j)** army worm; **(k)** aphids; **(l)** Potosiabre vitarsis; **(m)** peach borer.

**Figure 9** presents representative detection samples for each category, providing a clear visualization of the AMS-YOLO model’s effectiveness in maize pest identification. Only results in which the predicted category matches the corresponding ground truth label are shown, ensuring an accurate reflection of the model’s recognition capability.

**Figure 10:**
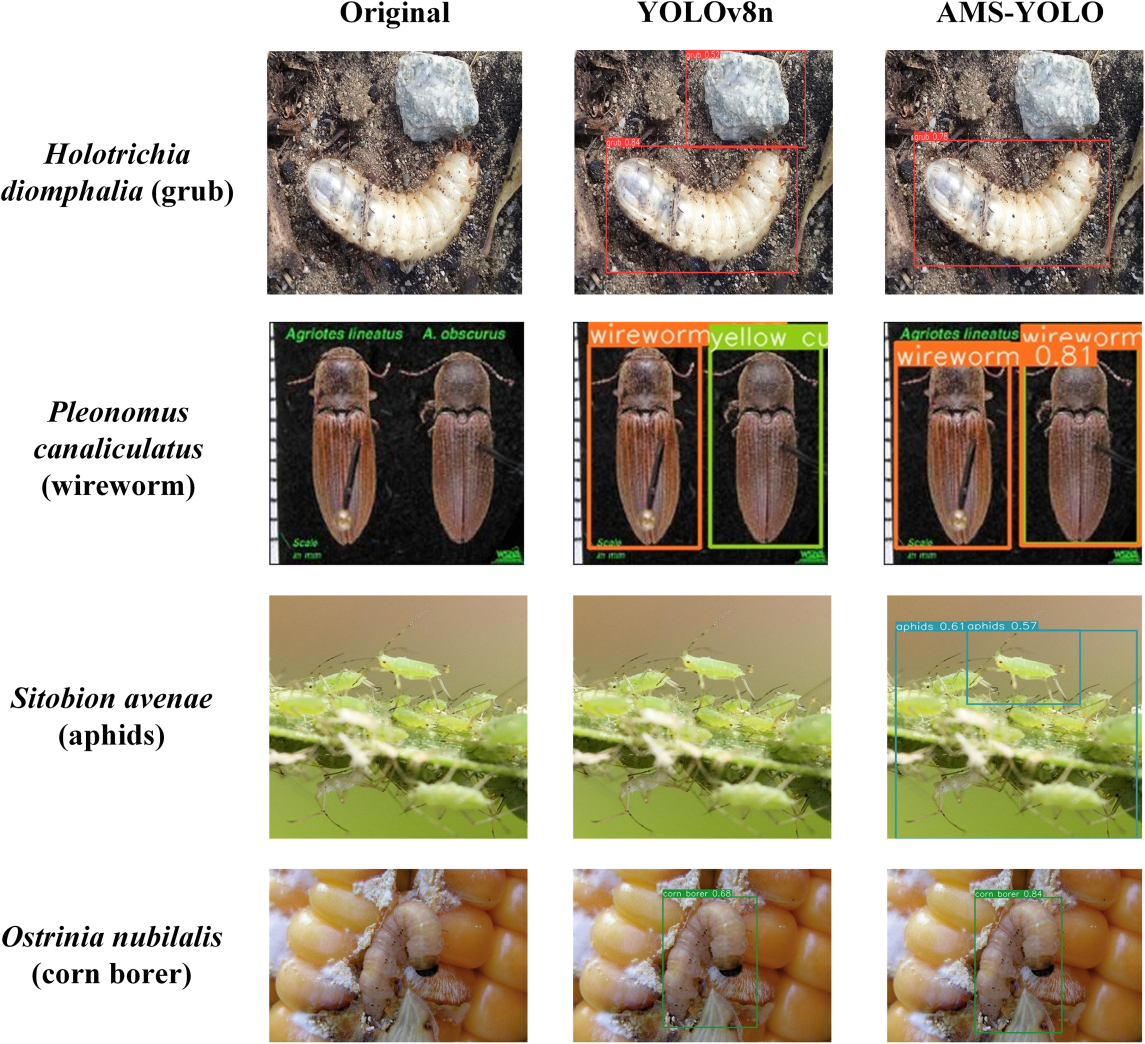
Detection result comparison between YOLOv8n and AMS-YOLO: left original images, middle YOLOv8n predictions, right AMS-YOLO predictions.

As shown in **Figure 10**, this study compares the visual performance of YOLOv8n and the improved AMS-YOLO model on identical pest images. For *Holotrichia diomphalia* detection, AMS-YOLO reduces false positives in background regions. When detecting *Pleonomus canaliculatus*, AMS-YOLO substantially decreases class confusion rates and eliminates false detections. For *Sitobion avenae* identification, AMS-YOLO successfully identifies individuals that YOLOv8n fails to detect. In *Ostrinia nubilalis* cases, AMS-YOLO maintains stable performance under complex backgrounds, achieving more precise bounding boxes. Results demonstrate that AMS-YOLO outperforms the baseline YOLOv8n model by reducing false positives, false negatives, and background interference while improving bounding box accuracy. These findings validate its potential for practical agricultural pest monitoring.

#### 4.3.2 Comparison of the effects of different attention mechanisms

To evaluate the effectiveness of SMCA, YOLOv8n was used as the baseline model, and different attention modules were embedded in C2f within the backbone. Compared attention mechanisms include ACMix (Pan et al., 2022), FLA (Han et al., 2023), iRMB (Zhang et al., 2023), EMA (Ouyang et al., 2023), and Triplet Attention (Misra et al., 2021). **Table 5** shows the effects of different attention modules on the model’s detection metrics.

**Table 5:** Performance comparison table of different attentions under multiple indicators.

Model	Precision (%)	Recall (%)	mAP50 (%)	mAP50:95 (%)	Parameters (M)
YOLOv8n	86.9	86.1	91.0	69.7	3.15
YOLOv8n+ACMix	86.3	87.7	91.6	69.6	3.12
YOLOv8n+FLA	87.8	87.5	91.7	70.1	3.12
YOLOv8n+iRMB	87.3	87.6	91.6	70.2	3.12
YOLOv8n+EMA	88.8	86.8	91.8	70.0	3.01
YOLOv8n+Triplet Attention	88.3	87.4	91.7	70.2	**3.01**
YOLOv8n+SMCA	**89.2**	**88.2**	**92.9**	**71.5**	3.12

Bold values represent the best comparison result for the corresponding metric.

The proposed SMCA module achieved optimal performance across all metrics. Precision was 89.2% and Recall was 88.2%, with mAP50 at 92.9%, showing significant improvements over other attention mechanisms. Precision improved by 2.9% compared to ACMix, indicating SMCA’s distinct advantage in separating maize pest features from complex backgrounds. Compared to FLA with similar parameters, SMCA improved Precision by 1.4%, demonstrating superior selectivity in feature extraction. With similar parameters, SMCA outperformed Triplet Attention mAP50 by 1.2%, showing the efficiency of the module design. The 71.5% improvement in mAP50:95 demonstrates that the model maintains strong performance across different detection thresholds, highlighting its practical applicability. These improvements demonstrate SMCA’s superiority in key morphological feature extraction. The module also excels in background interference suppression for maize pest detection.

#### 4.3.3 Comparison of performance at different locations of the attention mechanism

To evaluate the effectiveness of the SMCA mechanism in different model components of the model, this study embeds SMCA into both the Backbone and Neck structures of YOLOv8n and compares the resulting performance metrics. The experimental results are shown in **Table 6**:

**Table 6:** Comparison of performance of different locations.

Model	Precision (%)	Recall (%)	mAP50 (%)	mAP50:95 (%)	Parameters (M)
YOLOv8n	86.9	86.1	91.0	69.7	3.15
YOLOv8n-Neck-C2f	86.7	88.2	91.4	70.7	**3.09**
YOLOv8n-Backbone-C2f	**89.2**	**88.2**	**92.9**	**71.5**	3.12

Bold values represent the best comparison result for the corresponding metric.

These findings indicate placing SMCA in such Backbone offers significant advantages over Neck placement. Specifically, Backbone-C2f achieves 92.9% mAP50, marking a 1.9% improvement over original YOLOv8n, whereas Neck-C2f only achieves a 0.4% improvement. This performance difference occurs because the Backbone progressively extracts features from basic to advanced levels in the original image. By placing SMCA in the Backbone, the model can begin fusing local and global attention early in feature extraction. Additionally, C2f_SMCA in the Backbone handles multi-scale features ranging from 128 to 1024 channels. In contrast, it only processes limited P3, P4, and P5 scales in the Neck. This early-stage enhancement of multi-scale features enables better feature utilization in subsequent layers.

### 4.4 Ablation experiment

For verifying the performance of SMCA, AMConv, as well as MSBlock for improving YOLOv8n, we conducted ablation studies using the maize pest dataset. Eight models were tested, comparing performance between improved and original models across various metrics. **Table 7** presents the experimental results, analyzing the impact of different modules on the model’s performance improvement.

**Table 7:** Ablation study of AMS-YOLO on datasets.

Model	SMCA	AMConv	MSBlock	Precision(%)	Recall(%)	mAP50(%)	mAP50:95(%)	Parameters(M)	Weights(MB)
Model 1	×	×	×	86.9	86.1	91.0	69.7	3.15	6.3
Model 2	✓	×	×	89.2	88.2	92.9	71.5	3.12	6.25
Model 3	×	✓	×	87.8	87.1	92.5	71.1	2.59	5.2
Model 4	×	×	✓	89.6	86.8	92.3	71.1	2.84	5.8
Model 5	✓	✓	×	88.9	88.2	93.1	72.1	2.70	5.7
Model 6	✓	×	✓	87.2	87.4	92.2	71.0	2.95	6.0
Model 7	×	✓	✓	87.9	89.3	93.2	73.2	**2.42**	**5.0**
Model 8	✓	✓	✓	**90.0**	**89.8**	**94.2**	**73.7**	2.53	5.3

Bold values represent the best comparison result for the corresponding metric.

Model 2 shows that SMCA improves Precision from 86.9% to 89.2%, indicating effective reduction of false detections from background interference. Model 3 reveals that MSBlock increases Recall to 87.1% and mAP50:95 to 71.1%, proving strong adaptability in handling pests of different sizes. This proves particularly useful for simultaneous detection of pests at different developmental stages, such as adults and larvae. Model 4 indicates that AMConv reduces parameter count to 2.84M while maintaining high detection accuracy. This enables model deployment in real agricultural scenarios. Model 5 exhibits that SMCA and MSBlock synergy improves mAP50:95. The model enhances detection performance for varying target sizes while mitigating background interference. Models 6 and 7 demonstrate that multi-scale detection capability remains unaffected even with smaller parameters. Model 8 achieves optimal results, with 90.0% Precision and 89.8% Recall. This shows substantial progress in reducing false positives and negatives while maintaining small parameter size. mAP50 and mAP50:95 reach 94.2% and 73.7%, respectively.

**Figure 11:**
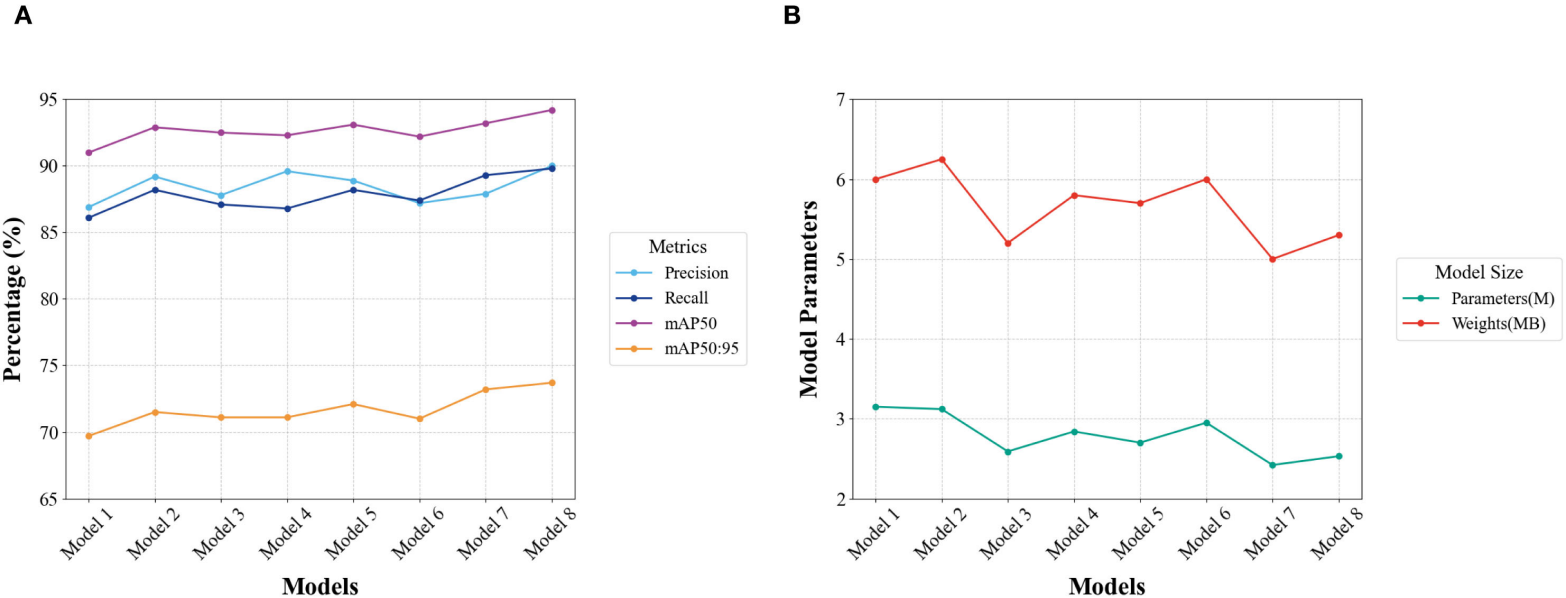
Trend curves of ablation experimental results. **(A)** Trend curves of detection Precision, Recall, mAP50, and mAP50:95 metrics for enhanced YOLOv8n implemented with SMCA, AMConv, and MSblock. **(B)** Trend curves showing the variations in Parameters and Weights metrics for the improved YOLOv8n model.

As shown in **Figure 11A**, incorporating SMCA, AMConv, and MSBlock consistently improves Precision, Recall, mAP50, and mAP50:95. **Figure 11B** demonstrates that these improvements are achieved with a reduced parameter count and more compact model size. This proposed approach achieves excellent balance between practicality and performance, confirming the synergistic effect of these three modules.

### 4.5 Comparison of other classical models

For additional validation of the advantages of the improved AMS-YOLO in maize pest detection, a comprehensive evaluation of 12 mainstream object detection models was conducted, including traditional architectures such as SSD, RetinaNet, RT-DETR, and various YOLO models. The evaluation metrics included Precision, Recall, mAP50, and mAP50:95, as well as model efficiency, Parameters, and Weights. The results are shown in **Table 8**.

**Table 8:** Comparison of different detection models.

Model	Precision (%)	Recall (%)	mAP50 (%)	mAP50:95 (%)	Parameters (M)	Weights (MB)
SSD	76.1	77.5	80.2	64.2	26.2	62.8
Retina Net	73.0	78.3	80.6	64.5	27.1	103.4
RT-DETR	89.9	87.6	88.2	69.7	42.8	160.3
YOLOv3-tiny	83.5	86.2	88.2	63.6	12.1	24.4
YOLOv5n	84.9	86.9	90.3	67.6	**2.50**	**5.3**
YOLOv5s	89.0	**89.9**	93.5	72.8	9.11	18.5
YOLOv6n	83.2	83.5	88.1	66.5	4.23	8.7
YOLOv8n	86.9	86.1	91.0	69.7	3.15	6.3
YOLOv9c	90.0	88.2	93.4	73.1	25.3	51.6
YOLOv10n	89.9	86.2	92.0	69.9	2.70	5.8
YOLOv11n	88.7	88.7	93.0	72.8	2.58	5.5
YOLOv12n	86.2	86.6	91.0	69.0	2.51	5.4
AMS-YOLO	**90.0**	89.8	**94.2**	**73.7**	2.53	5.5

Bold values represent the best comparison result for the corresponding metric.

AMS-YOLO demonstrates superior performance across all detection metrics while maintaining lightweight design. Compared with lightweight YOLO variants like YOLOv5n and YOLOv12n, AMS-YOLO achieves advanced metrics with comparable parameter counts, indicating optimal balance between performance and computational efficiency. Compared with traditional architectures like SSD and RetinaNet, AMS-YOLO reduces computational overhead while improving detection performance by 15%. Transformer-based RT-DETR exhibits strong performance but requires 42.8M parameters. In contrast, AMS-YOLO achieves superior detection results with only 2.53M parameters, highlighting the proposed method’s efficiency.

**Figure 12:**
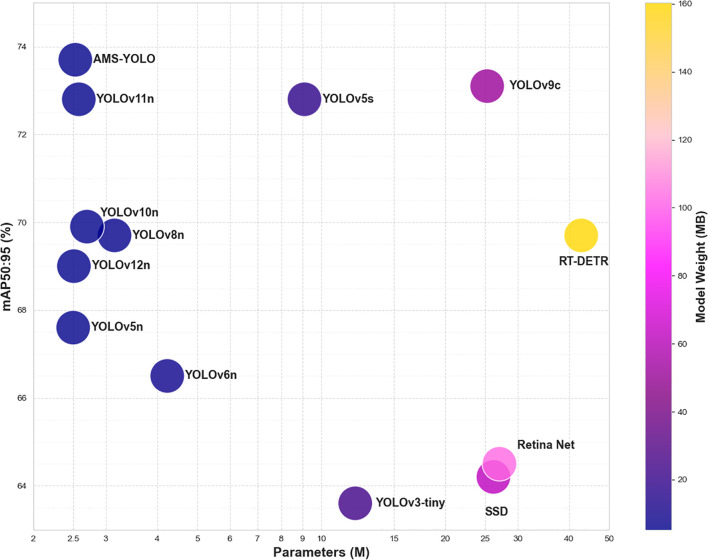
Comparison of object detection model performance: mAP50:95, parameter and model weight (MB) bubble diagram.

As illustrated in **Figure 12**, the scatter plot clearly demonstrates the trade-off between model complexity Parameters and mAP50:95, with color representing model weight.

### 4.6 Edge device deployment for sustainable pest monitoring

**Figure 13:**
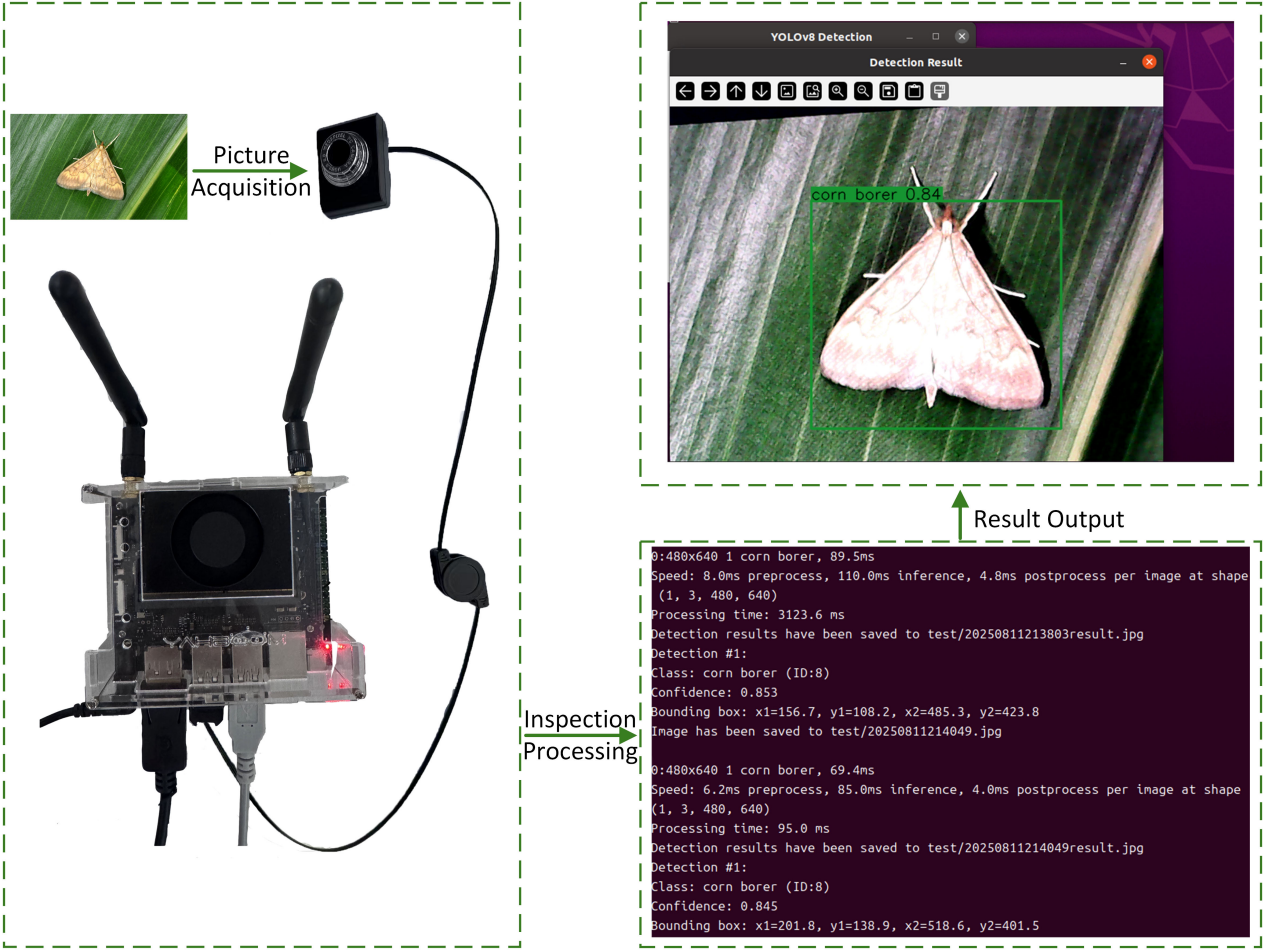
Real-time detection system and deployment workflow.

During evaluation, we deployed the trained AMS-YOLO model on the NVIDIA Jetson Nano embedded development board. This provided a cost-effective intelligent monitoring solution for sustainable plant protection. The device featured CUDA support to accelerate inference and meet real-time field monitoring requirements. The system captured images at 480×640 pixel resolution through an external camera. This enabled pest detection in natural farmland environments. All experiments used simulated field conditions to ensure result applicability in real-world agricultural settings. **Figure 13** shows the real-time detection system and deployment workflow, demonstrating the complete process from image acquisition to pest identification results on the target hardware platform.

As shown in **Table 9**, during stable operation, YOLOv8n requires 85.0 ms for inference, with total processing time of 95.0 ms at 10.52 FPS. In contrast, AMS-YOLO reduces inference time to 69.4 ms and total processing time to 81.7 ms, achieving 12.25 FPS. AMS-YOLO improves inference performance by 18.4%, overall processing efficiency by 14.0%, and frame rate by 16.4%. Resource utilization analysis reveals that AMS-YOLO achieves 58.5% CPU utilization during warm-up, markedly lower than YOLOv8n’s 74.7%, demonstrating enhanced resource efficiency through architectural optimization. Lower CPU utilization reduces device heat generation, extends battery life, and decreases overall power consumption.

**Table 9:** Inference time breakdown and system performance.

Model	Test run	Preprocessing time (ms)	Inference time (ms)	Postprocessing time (ms)	Total processing time (ms)	FPS	Max CPU percent (%)
YOLOv8n	Warm-up Phase	8.0	110.0	4.8	3123.6	0.32	74.7
	Stable Operation Phase	6.2	85.0	4.0	95.0	10.52	
AMS-YOLO	Warm-up Phase	7.3	89.5	4.2	3033.7	0.32	58.5
	Stable Operation Phase	5.3	69.4	3.2	81.7	12.25	

Experimental results demonstrate that AMS-YOLO effectively improves inference efficiency through architectural optimization. This proves critical for field edge monitoring devices powered by solar panels or limited battery systems. The approach provides cost-effective intelligent monitoring solutions for sustainable plant protection.

### 4.7 Interpretability experiment

**Figure 14:**
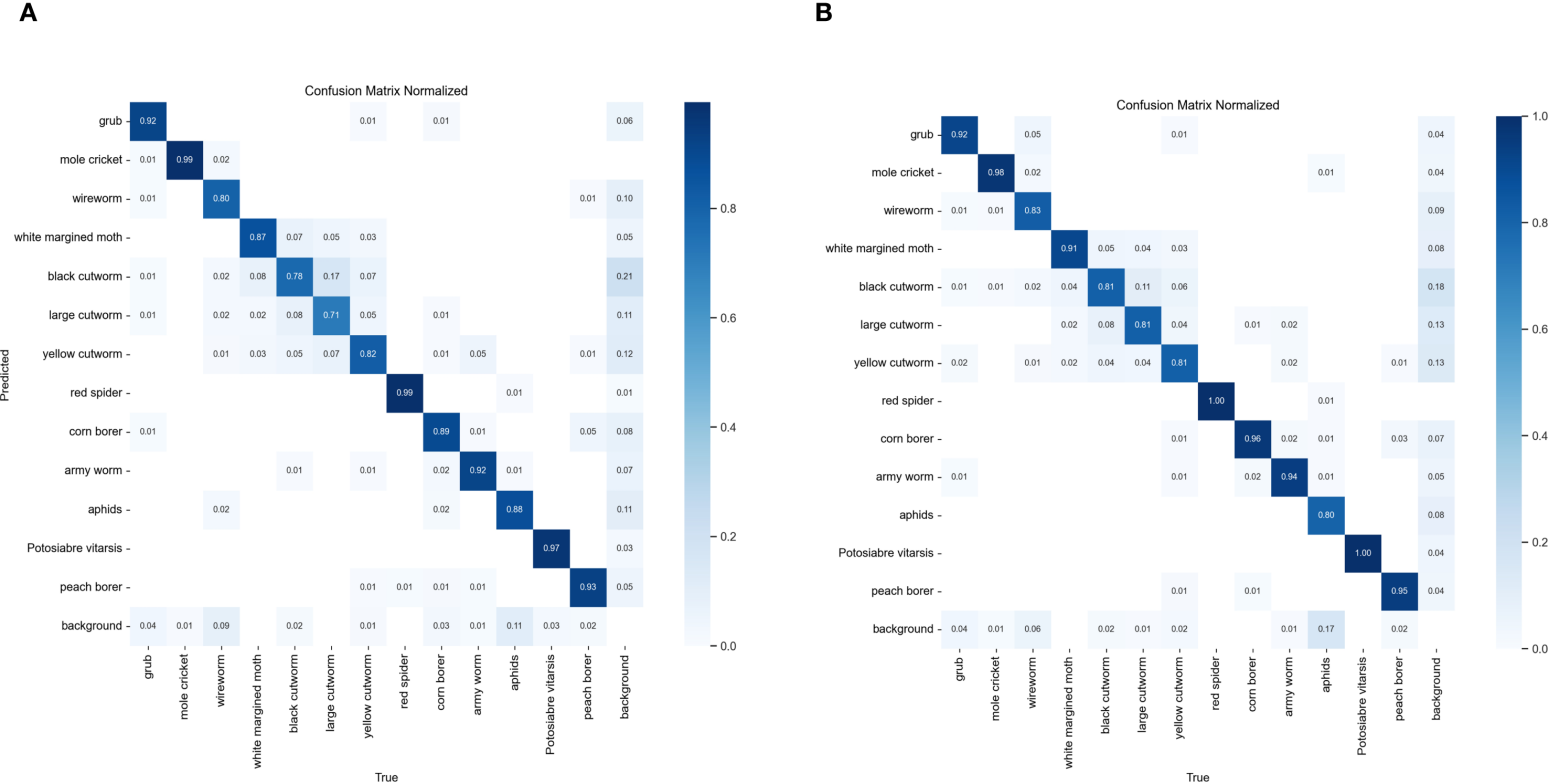
Confusion matrix visualization of YOLOv8n and AMS-YOLO models for agricultural pest detection. **(A)** YOLOv8n baseline model confusion matrix. **(B)** The proposed AMS-YOLO model confusion matrix.

The confusion matrix provides clear representation of model predictions versus true labels. It illustrates classification performance for each category, including TP, FP, TN, and FN. This matrix offers critical insights for comprehensive model performance evaluation. **Figure 14** shows the confusion matrices for AMS-YOLO and YOLOv8n models on this dataset.

Quantitative analysis based on the normalized confusion matrix shows that our improved method outperforms the YOLOv8n baseline model in multi-class pest recognition tasks. For example, black cutworm recognition accuracy improved from 78% to 81%. The confusion rate with large cutworm decreased substantially from 17% to 11%. This improvement demonstrates that fine-grained feature extraction improved considerably. The model better captures subtle differences between nocturnal moth pests, exhibiting stronger discriminative power in classifying similar species. For aphid classification, the misclassification rate dropped markedly from 11% to 7%, a 36.4% reduction. This indicates the model is more robust against complex background interference and more accurately distinguishes target pests from background environment.

**Figure 15:**
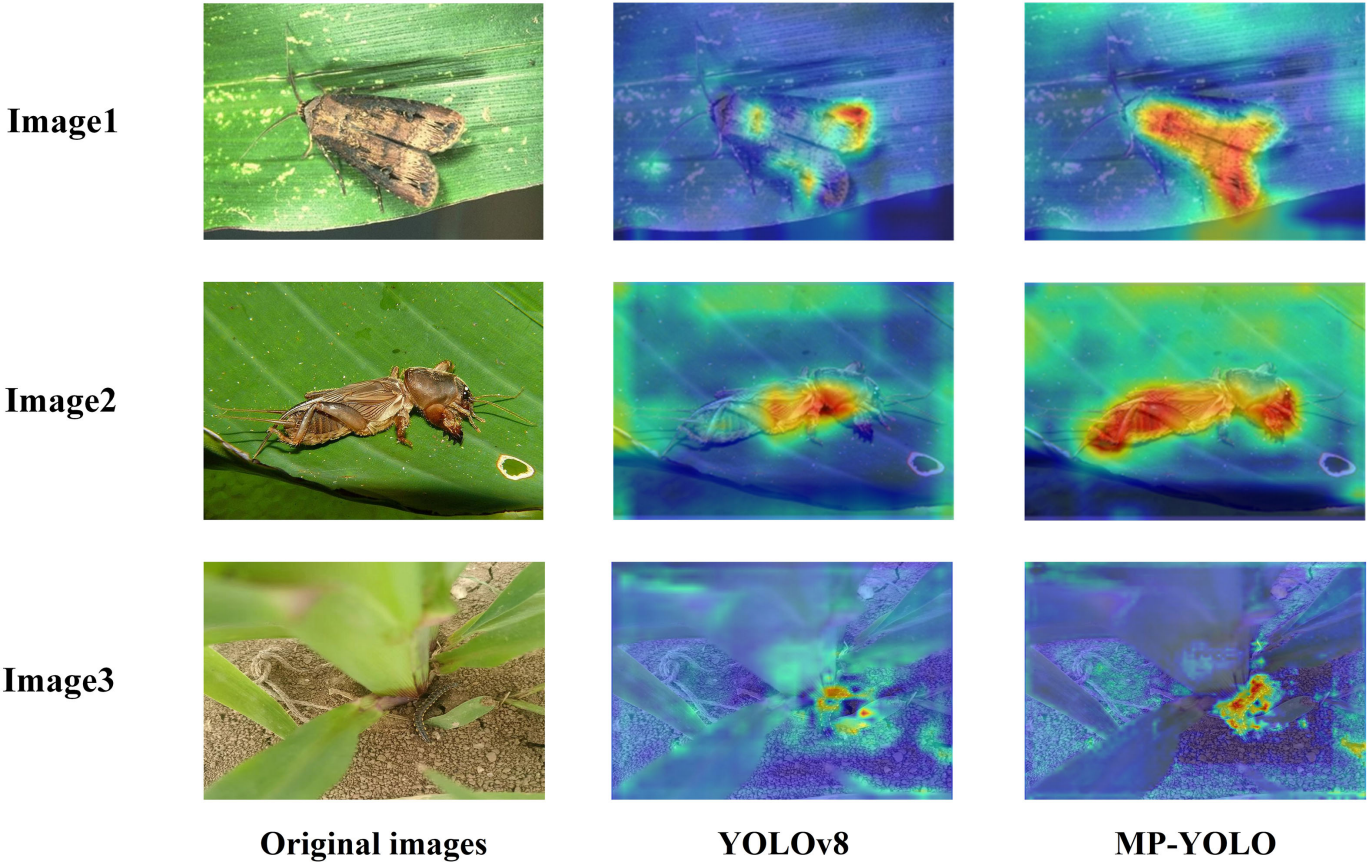
YOLOv8n and AMS-YOLO heat map visualization comparison, image1, image2 and image3 are examples of black cutworm and mole cricket respectively.

**Figure 15** shows heatmap comparison of YOLOv8n and AMS-YOLO models. The more pronounced and complete red region coverage demonstrates that AMS-YOLO exhibits more focused attention distribution across the insect’s body. This indicates that the model effectively reduces background interference while enhancing its ability to capture spatial details.

## 5 Discussion

The proposed AMS-YOLO model achieved 94.2% mAP50 and 73.7% mAP50:95, improving by 3.2% and 4.0% over baseline YOLOv8n. Integrating SMCA attention mechanism, AMConv downsampling, and MSBlock multi-scale fusion enhances detection accuracy under challenging agricultural conditions. Our approach addresses limitations in traditional methods. Manual identification suffers from subjectivity and scalability issues. Image processing methods using handcrafted features demonstrate poor adaptability to complex agricultural environments. Traditional machine learning approaches like SVM and Random Forests rely on manual feature design, limiting performance on high-dimensional agricultural imagery.

Attention mechanisms have proven valuable in agricultural pest detection applications. Recent developments include CSE-ELAN for soybean pest detection (Chen et al., 2025) and MDGA for litchi disease identification (Li et al., 2025), highlighting specialized attention designs in complex agricultural contexts. Our SMCA module builds upon these advances by effectively addressing complex background interference while excelling in differentiating morphologically similar pest species. This is achieved through strategic combination of spatial attention computation with multi-level contextual attention mechanisms. The synergistic effect of AMConv and MSBlock modules further optimizes performance: AMConv preserves critical information during downsampling through its dual-path architecture, while MSBlock enhances the model’s capability to identify pests across different developmental stages through multi-scale feature fusion. Edge deployment testing on NVIDIA Jetson Nano validated real-world applicability, demonstrating robust computational performance with real-time detection at 12 FPS. This performance establishes technical feasibility for continuous farmland monitoring, marking an important step toward practical deployment in agricultural settings.

Several limitations warrant acknowledgment. First, our dataset encompasses only 13 maize pest species, potentially limiting generalizability to broader pest populations. Second, while 12 FPS performance on Jetson Nano represents a significant achievement, the model still demands higher computational resources and power consumption than traditional image processing approaches. Third, model robustness requires further validation under extreme environmental conditions, such as intense direct sunlight or heavy rainfall. Despite notable improvements in distinguishing morphologically similar species, misidentification risks persist when interspecies morphological differences are minimal. Finally, model generalization across different geographical regions and seasonal variations needs validation through more extensive field trials.

Future research should prioritize three critical areas to advance this technology toward widespread adoption. First, developing large-scale, standardized pest datasets is essential to improve model generalization, as pest morphological characteristics vary considerably due to geographical location, climatic conditions, host plant variations, and nutritional status (Liu and Wang, 2021). Second, deep optimization of lightweight technologies represents a crucial step toward achieving true edge intelligence. Building on our successful edge deployment experience, future efforts should focus on systematic optimization strategies, including advanced model pruning and knowledge distillation (Xu et al., 2024; Zhang et al., 2024). Third, integrating IoT and AI technologies will be instrumental in building comprehensive intelligent monitoring ecosystems. This technological convergence can facilitate a paradigm shift from reactive pest management to proactive prevention strategies through data-driven decision support systems (Ahmed et al., 2024; Kariyanna and Sowjanya, 2024).

These technological advances will establish the foundation necessary to translate our theoretical contributions into practically deployable intelligent monitoring systems. By providing robust technical support for sustainable plant protection objectives, this work contributes to promoting harmonious agricultural-environmental development in modern farming systems.

## 6 Conclusions

Within this study, we propose a lightweight maize pest detection model upon the YOLOv8n algorithm, named AMS-YOLO. First, the proposed SMCA module replaces the C2f feature extraction module in the backbone. This module effectively addresses confusion among similar pests while suppressing background interference from maize leaves and stalks. Second, AMConv replaces traditional convolution for downsampling, reducing feature map size while preserving key information and reducing computational burden. Finally, MSBlock replaces the original C2f module in the neck for feature extraction. This solves scale inconsistency problems in pest detection and enhances key localization information.

Through extensive validation experiments, AMS-YOLO demonstrates excellent performance. Compared with the original model, AMS-YOLO improves mAP50 by 3.2%, mAP50:95 by 4%, Precision by 3.1%, and Recall by 3.7%. The model requires only 2.53M parameters and 5.5MB storage. Comparative experiments further demonstrate that AMS-YOLO outperforms several widely used target detection models, including SSD, RetinaNet, RT-DETR, and various YOLO families. Actual deployment tests demonstrate that AMS-YOLO maintains stable real-time monitoring capabilities in resource-constrained environments. This provides reliable support for precise prevention and control decisions. Therefore, the AMS-YOLO model suits maize pest detection tasks well. Its compact size provides a reliable reference solution for edge computing devices.

This study promotes object detection algorithm applications in agriculture. It provides practical tools for sustainable and intelligent plant protection, demonstrating computer vision technology’s critical value in supporting sustainable agricultural development. As a key component of intelligent agricultural infrastructure, these results provide strong support for precision agriculture development. The results are expected to contribute positively to building more environmentally friendly and efficient modern agricultural production systems. Additionally, they help promote agricultural green transformation and intelligent infrastructure construction.

## Data Availability

The original contributions presented in the study are included in the article/supplementary material. Further inquiries can be directed to the corresponding author.
